# An Immunocompetent Patient with a Vesicular Rash and Neurological Symptomatology

**DOI:** 10.1155/2013/168943

**Published:** 2013-12-03

**Authors:** Chad J. Cooper, Sarmad Said, Mohamed Teleb, Paola Rosa, S. Claudia Didia

**Affiliations:** Department of Internal Medicine, Texas Tech University Health Science Center, 4800 Alberta Avenue, El Paso, TX 79905, USA

## Abstract

Viral infection is the most common cause of aseptic meningitis with the most frequent virus associated with aseptic meningitis being enteroviruses (coxsackievirus and echovirus). In viral meningitis, cerebrospinal fluid (CSF) shows a mild pleocytosis with a lymphocytic predominance, elevated protein, and normal glucose level. Nucleic acid amplification methods have greatly improved the detection of viral pathogens. In our case, a 47-year-old Caucasian female patient presented with a persistent throbbing headache for six days, localized at the frontal area, associated with photophobia, and exacerbated by bright lights and loud noises. Physical examination revealed nuchal rigidity and a vesicular rash at the right T4–T6 dermatome region. CSF findings were consistent with aseptic meningitis and polymerase chain reaction (PCR) was positive for VZV. Clinical improvement in meningeal signs and symptoms occurred after the initiation of acyclovir to complete a total 10-day course. There are no published data revealing that acyclovir will modify the course of VZV meningitis, but it is important to recognize the potential clinical benefit with the early initiation of antiviral therapy, especially if a zoster rash is discovered on examination. However, this is rarely the case because the majority of VZV meningitis will not present with a rash. Even though the reactivation of VZV is not usually associated with clinical meningitis, it is important to consider VZV in the differential diagnosis of a patient presenting without a rash with CNS disease. PCR has been proven to be a useful and quick diagnostic tool in the early diagnosis of VZV-associated neurological disease.

## 1. Introduction

Aseptic meningitis will frequently have a similar presentation to bacterial meningitis such as fever, headache, altered mental status (AMS), stiff neck, and photophobia. In the majority of patients with aseptic meningitis, the clinical course will be self-limited and eventually will be resolved without specific therapy. AMS is an important distinguishing feature between encephalitis and meningitis. Viral infection is the most common cause of aseptic meningitis with the most frequent virus associated with aseptic meningitis being enteroviruses (coxsackievirus and echovirus). Other viral infections that can lead to aseptic meningitis include mumps, arbovirus, measles, influenza, human immunodeficiency virus (HIV), West Nile virus (WNV), and herpes viruses such as herpes simplex virus, Epstein-Barr virus, and varicella-zoster virus, or in rare cases lymphocytic choriomeningitis virus [1].

A detailed history and physical examination can provide a clue to the etiologic diagnosis. In viral meningitis, CSF will show a mild pleocytosis with a lymphocytic predominance, elevated protein, and normal glucose level. Nucleic acid amplification methods such as PCR have greatly improved the detection of viral pathogens. Magnetic resonance imaging (MRI) should be considered for any patient who presents with clinical signs or symptoms suggesting encephalitis.

Skin manifestations may suggest the diagnosis of aseptic meningitis from certain causes. Examples include a vesicular rash of varicella zoster, the genital lesions of HSV-2, or a mild maculopapular rash occurring in summer and fall months with some enteroviruses. The potential manifestations of VZV reactivation include herpes zoster, postherpetic neuralgia, aseptic meningitis and encephalitis, Ramsay Hunt syndrome, and herpes ophthalmicus. Herpes zoster is the most common presentation of reactivated VZV with the majority of cases occurring in adults. Patients will typically report a prodromal illness of pain, pruritus, burning, or paresthesias. Over the course of a few days, erythematous macules and papules develop along a single dermatome progressing to vesicles that eventually crust and heal over. Simultaneous involvement of the brain, spine and, meninges in response to VZV reactivation is rare in general except in cases of immunocompromised patients [2]. Previous cases of varicella-zoster virus meningitis have been reported, but in this particular case the appearance of the vesicular rash was present during the neurological symptomatology rather than a later finding after patient condition had already improved.

## 2. Case Presentation

Forty-seven-year-old Caucasian female patient presented with a persistent throbbing headache for six days, localized at frontal area, associated with photophobia, and exacerbated by bright lights and loud noises. She denied any sick contacts or recent travel. She had a low-grade fever of 37.6 degrees celsius. She had a single episode of nausea and vomiting on the day of admission. She described the feeling of being dizzy for 1 day as if she was going to lose her balance. She also had an intermittent ringing sensation in both ears and suffered fatigue for the past 2 days. She did not complain of any confusion, memory loss, loss of hearing or vision, changes in personality, tremors, or seizures. Patient claims that no rash was present prior to admission. She has a past medical history of hypertension.

**Figure 1:**
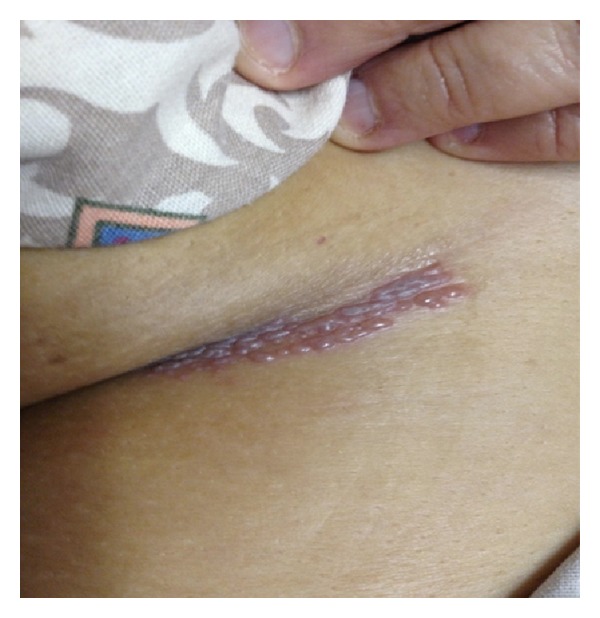
The skin involvement characterized through vesicular rash extending over the dermatome T4–T6.

Initial vital signs on admission were normal except for stage 2 hypertension (161/79 mmHg). Pertinent physical examination findings were that she was alert but in mild distress secondary to pain and photophobia. Her pupils were 3 mm bilaterally, equal, round, and reactive to light with intact extraocular movements and no nystagmus. Tympanic membrane examination was assessed and no abnormalities were noted. Observation of the skin revealed a painful vesicular rash at the right T4–T6 dermatome region near the right breast as demonstrated in Figure 1. All cranial nerves were assessed and normal on examination. The Rinne test revealed air conduction that is better than bone conduction and the Weber test showed equal lateralization to both ears. No motor or sensory deficits to light touch, pain, position sense, or vibration are noted. All reflexes such as biceps, knee tendon, and Babinski sign were normal. No deficits were in muscle strength (5/5 all four extremities) or tremors. Nuchal rigidity was observed but Kernig's and Brudzinski's signs were negative.

Initial laboratory work-up was unremarkable. CSF assessment revealed colorless and clear fluid with WBC of 648 per uL and lymphocytes of 92%, RBC of 47 per uL, protein 396 mg/dL, glucose 41 mg/dL, sodium 151 mmol/L, and LDH per uL. The serum glucose at the time of lumbar puncture was 56 mg/dL. CSF antigens were negative for group *B streptococcus*, *haemophilus*, *S*. *pneumonia*, and *N*. *meningitidis*. The HIV screening test was negative. Other CSF results included a coccidioides antibody in CSF, herpes-simplex-PCR, and West Nile virus IgM and IgG, which all were negative.

Empiric therapy of vancomycin 1 g IV BID, ceftriaxone 2 g IV daily, and dexamethasone 10 mg IV every 6 hours and acyclovir 800 mg PO every 4 hours were initiated upon admission. The infectious disease team was consulted and agreed with initial management and likely etiology of VZV meningitis. The antibiotic regimen and dexamethasone were discontinued after CSF cultures were negative for 48 hours. On the second hospital day, her clinical symptoms had significantly improved and she did not display any neurological deficits. The PCR for VZV in CSF was positive by the third day. The CSF culture, CSF fungal culture, blood culture, and acid-fast-bacilli culture were all negative. The electroencephalogram, CT brain, and MRI brain were all unremarkable. The patient was discharged on the third hospital day with instructions to continue acyclovir 800 mg PO every 4 hours for 7 more days. The patient had followup at the internal medicine clinic after one month with no neurological deficits or confusion.

## 3. Discussion

The specific therapeutic approach to a patient with viral meningitis will depend upon the clinical presentation of the patient and the existence of underlying factors. Empiric therapy with antibiotics for 48 hours in addition to acyclovir should be considered even if viral meningitis is suspected, in the elderly, immunocompromised. Otherwise, it is reasonable to observe the clinical status of the patient without antibiotic therapy. Enterovirus infection is the most common cause of viral meningitis in immunocompetent adult [3]. There are no published data revealing that acyclovir will modify the course of VZV meningitis, but it is important to recognize the potential clinical benefit with the early initiation of antiviral therapy, especially if a zoster rash is discovered on examination [4]. The reactivation zoster rash of VZV is seldom associated with aseptic meningitis. The clinical implications are important because VZV meningitis may not be considered in the differential diagnosis of a patient presenting without a rash [5]. HSV-2 and VZV neurological infections are associated with a higher CSF protein level compared to enteroviral infection [6]. The PCR is a quick diagnostic tool for the early diagnosis of VZV-associated neurological disease. The clinical diagnosis of VZV meningitis is easily thought of with the appearance of the typical zoster rash, however, in the absence of vesicular eruptions, the PCR assay can be a valuable tool [7]. VZV meningitis should always be considered in the differential diagnosis of a clinical presentation of aseptic meningitis; however, with the appearance of the typical zoster rash, the suspicion is elevated, but in the majority of cases the zoster rash never occurs or appears later when the clinical status has already improved.
